# Analysis of Essential Arabidopsis Nuclear Genes Encoding Plastid-Targeted Proteins

**DOI:** 10.1371/journal.pone.0073291

**Published:** 2013-09-04

**Authors:** Linda J. Savage, Kathleen M. Imre, David A. Hall, Robert L. Last

**Affiliations:** 1 Department of Biochemistry and Molecular Biology, Michigan State University, East Lansing, Michigan, United States of America; 2 Department of Plant Biology, Michigan State University, East Lansing, Michigan, United States of America; Kansas State University, United States of America

## Abstract

The Chloroplast 2010 Project (http://www.plastid.msu.edu/) identified and phenotypically characterized homozygous mutants in over three thousand genes, the majority of which encode plastid-targeted proteins. Despite extensive screening by the community, no homozygous mutant alleles were available for several hundred genes, suggesting that these might be enriched for genes of essential function. Attempts were made to generate homozygotes in ∼1200 of these lines and 521 of the homozygous viable lines obtained were deposited in the Arabidopsis Biological Resource Center (http://abrc.osu.edu/). Lines that did not yield a homozygote in soil were tested as potentially homozygous lethal due to defects either in seed or seedling development. Mutants were characterized at four stages of development: developing seed, mature seed, at germination, and developing seedlings. To distinguish seed development or seed pigment-defective mutants from seedling development mutants, development of seeds was assayed in siliques from heterozygous plants. Segregating seeds from heterozygous parents were sown on supplemented media in an attempt to rescue homozygous seedlings that could not germinate or survive in soil. Growth of segregating seeds in air and air enriched to 0.3% carbon dioxide was compared to discover mutants potentially impaired in photorespiration or otherwise responsive to CO_2_ supplementation. Chlorophyll fluorescence measurements identified CO_2_-responsive mutants with altered photosynthetic parameters. Examples of genes with a viable mutant allele and one or more putative homozygous-lethal alleles were documented. RT-PCR of homozygotes for potentially weak alleles revealed that essential genes may remain undiscovered because of the lack of a true null mutant allele. This work revealed 33 genes with two or more lethal alleles and 73 genes whose essentiality was not confirmed with an independent lethal mutation, although in some cases second leaky alleles were identified.

## Introduction

Chloroplasts perform diverse functions in plant metabolism, growth and development, ranging from photosynthetic carbon fixation, through hormone metabolism, to production of core metabolites including protein amino acids and vitamin cofactors. Despite years of analysis of plastid biochemistry and physiology, functions are reported for a relatively small fraction of the plastid proteome. Much of what is known about plastid function comes from analysis of mutants defective in plastid-targeted proteins; however lethality of mutants in essential functions limits the utility of genetics to a subset of plastid processes [1]–[4].

Screening for mutations that lead to homozygous seed or seedling lethality is an effective approach to identifying essential genes [5]–[7]. In a limited number of cases, homozygotes can be partially rescued for study if specific conditions required for survival are met, such as increased CO_2_ to suppress photorespiration [8], supplying germinating seedlings with sucrose [9], or supplementation with a missing nutrient such as an amino acid [10], [11] or vitamin [12].

While many viable mutants defective in function of chloroplast proteins were described in plants [13]–[17], large-scale targeted efforts to identify essential nuclear genes encoding chloroplast proteins were only recently published [1], [6], [18]. As part of a large effort to characterize *Arabidopsis thaliana* mutants defective in several thousand nuclear-encoded genes predicted to produce plastid-targeted proteins, the Chloroplast 2010 Project (http://www.plastid.msu.edu/) sought to identify phenotypes of >5000 T-DNA mutants. The general approach was to identify genes known or predicted to encode plastid-targeted proteins and phenotypically analyze confirmed homozygous insertion mutants [19]–[22]. As expected for mutations that knock out gametophyte function, impact seed or seedling development, or prevent the plant from surviving to reproductive phase, no homozygous mutants became available for several hundred target genes. This work describes attempts to identify homozygous mutant lines for these genes and characterization of seedling mutant phenotypes of those alleles that failed to yield viable homozygotes in soil. We describe mutants defective at various stages of seed development, and mutants with defects in seedling development that responded to supplementation with sucrose, amino acids or CO_2_ enrichment. The collective results add annotation to >200 publically available *A. thaliana* mutants. These include 33 genes with two independent seed or seedling development defective mutants, 26 with both strong amorphic seed or seedling development and leaky hypomorphic alleles, and 36 with a single seed or seedling development defective allele. This study also led to submission of 521 homozygous mutants and 128 seed stocks segregating for lethal alleles to the Arabidopsis Biological Resource Center (ABRC; http://www.arabidopsis.org).

## Results and Discussion

### Targeting Potential Homozygous Lethal Mutants

**Figure 1:**
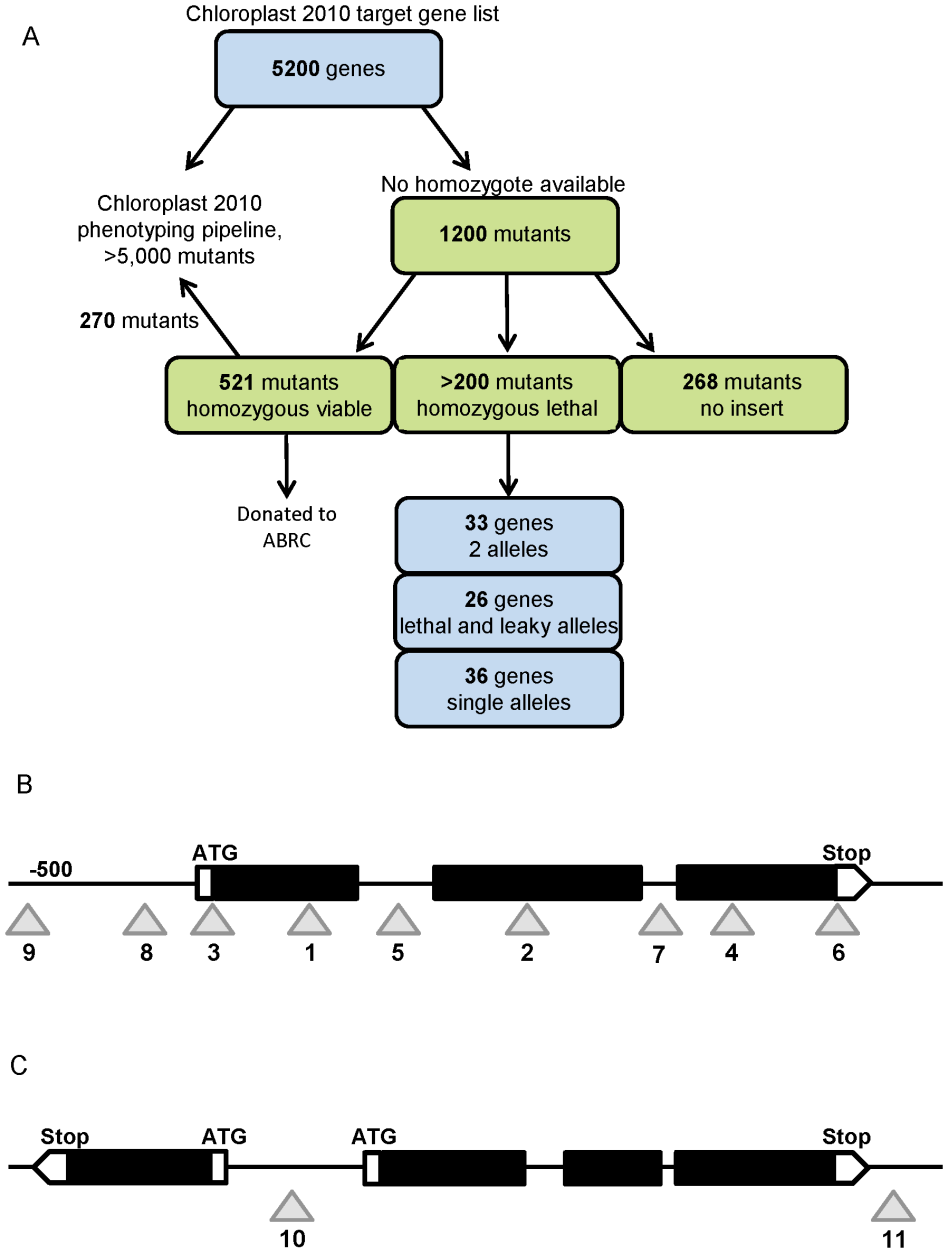
Project workflow, and allele strength scoring scheme based on likelihood of the T-DNA insertion eliminating gene expression. A. Project workflow starting from the list of chloroplast-targeted genes (top) through mutants analyzed to a summary of the lethal genes, (bottom). B and C, description of scoring schemes used to evaluate mutant alleles. Black boxes represent exons, thin solid lines show introns or non-coding regions, white boxes indicate 5′ (ATG) and 3′ (Stop) ends of the protein coding region, gray triangles represent T-DNA insert location, with numerical “strength” score below. B: 1, exon in first third of open reading frame (ORF); 2, exon in second third of ORF; 3, at extreme 5′ end of first exon; 4, exon, last third of ORF; 5, intron, first half of ORF; 6, at far 3′ end of last exon; 7, intron, last half of ORF; 8, 5′ non-coding region within 500 base pairs; 9, 5′ non-coding region >500 base pairs. C: 10, between two divergently transcribed genes; 11, 3′ non-coding region.

The target gene list first developed in 2004 for the Chloroplast 2010 project was used as the starting place for this study [19], [23] (http://www.plastid.msu.edu/). This included >5,200 nuclear *Arabidopsis thaliana* genes identified as putatively producing chloroplast-targeted proteins based on bioinformatic analysis of gene models in The Arabidopsis Information Resource (TAIR; http://www.arabidopsis.org) as well as from various lines of experimental evidence collated in SUBA (*SUB*cellular location database for *A*rabidopsis proteins; Heazlewood et al., 2007) and the Plant Proteome Database [24]. Current experimental or bioinformatic evidence for chloroplast localization curated at SUBA and the Plant Proteome Database for the characterized mutants is included in Tables 1 and S1. The goal of the project was to create phenotypic annotation for as many of these genes as possible. Publications and public domain databases describe previously characterized homozygous lethal mutants (http://www.seedgenes.org/, http://rarge.psc.riken.jp/phenome/, http://www.arabidopsis.org), and mutants defective in these genes were avoided in the current study. The project workflow beginning from this gene target list is summarized in Figure 1A.

**Table 1:** Mutants with defects affecting seedling development.

Annotated locus	Alleles	Allele score^a^	Chloroplast localization evidence	Phenotype	Media screen results^b^	High CO_2_ phenotype^c^	CF phenotype^d^
At1g71720^e^	SAIL_162_G11	2	experimental, [72], [73]	seedling lethal	4b	no	ND^f^
At2g28390	SALK_075382	1	TargetP score 837 stroma^g^	seedling lethal	1	no	ND
	SALK_039520	7		seedling lethal	1	no	ND
At2g39080^e^	SALK_006849	1	experimental, [74], [75]	seedling lethal	2	no	ND
At3g12290	SALK_015165	2	experimental, [75]	severe survival or growth defect	5	no	ND
	SALK_039538	5		severe survival or growth defect	5	no	ND
	WiscDsLox244C04	5		severe survival or growth defect	5	no	ND
At3g12590	SALK_044513	4	TargetP score 962 stroma	severe survival or growth defect	4c	yes	no
	SALK_129651	5		severe survival or growth defect	4c	no	no
At3g55800 [76]	SALK_130939	4	experimental, [72], [75], [77]–[80]	severe survival or growth defect	5	no	ND
	SALK_090549	5		severe survival or growth defect	5	no	ND
At4g14605 [81]	SAIL_425_E03	2	experimental, [63]	severe survival or growth defect	2	no	yes
	SALK_097243	4		severe survival or growth defect	2	yes	yes
At4g17620	SALK_103157	1	TargetP score 915 stroma	severe survival or growth defect	4c	yes	yes
	SALK_032903	5		severe survival or growth defect	4c	yes	yes
At4g17740	WiscDsLoxHS194_10B	5	experimental, [72], [75], [77], [79], [82]	seedling lethal	4b	no	no
	SALK_056011	8		seedling lethal	4b	no	no
At4g35250 [83]	SALK_058477	1	experimental, [75], [77], [78]	seedling lethal	2	no	ND
	SAIL_181_B09	7		seedling lethal	ND	no	ND
At5g04920^e^	SALK_130246	1	TargetP score 588 stroma	seedling lethal	2	no	ND
At3g17930 [51]	SALK_088638	4	experimental, [84], [85]	seedling lethal	4b	yes	yes
	FLAG_202E10	3		small morphology	4b	no	yes

a See Figure 1.

b See Figure 4 and Table S6.

c See Table 2, Figure S3 and Table S7.

d See Table 3.

e Gene has second allele with seed phenotype: At1g71720, mutant SALK_107226, mild seed pigment defective; At2g39080, mutant SALK_087872, developing seed defect; At5g04920, mutant SALK_131534, developing seed defect.

f ND, not done.

g Bioinformatic evidence is given for genes with no experimental evidence.

Several strategies were used to identify new homozygous lethal mutations. First, genotyping information from the SALK T-DNA genotyping project was evaluated [25] (http://signal.salk.edu/gabout.html) to find genes for which no homozygous mutant alleles were found. The hypothesis was that the set of alleles not found to yield homozygotes in the Salk Institute group’s genotyping of up to 16 progeny would be enriched for those causing lethality or poor seed set under standard growth conditions. Second, as thousands of putative homozygous mutant alleles were genotyped in the Chloroplast 2010 phenomics project [19], our own genotyping data set was searched for alleles without homozygous mutants. Once candidates were identified, annotated T-DNA insert location was evaluated (Figure 1B): genes with homozygous mutants with T-DNA insertions in locations less likely to completely disrupt gene expression, such as the 5′ UTR (scores 8 or 9, Figure 1B), but no homozygotes for alleles in exons (scores 1, 2, 3, or 4, Figure 1B), were considered noteworthy. Other large collections of T-DNA mutants such as GABI-KAT [26] and individual community-deposited mutants were also searched for candidates.

The list of tested mutants was structured to increase the chance for discovery of novel homozygous lethal mutants. Genes with uninformative annotation, which had compelling computational or experimental plastid targeting information documented in SUBA [27], were prioritized. Certain classes of uncharacterized predicted gene products were also chosen. For example, pentatricopeptide repeat proteins were targeted since examples of essential functions for genes encoding this protein motif were previously reported in a variety of plant species [28]–[31]. Known homozygous lethal mutants were included as assay controls to validate and help interpret results for the unknowns. To aid in interpreting the data, assay results for both the previously characterized controls and newly targeted mutants are included in the supplemental tables. If, during our experiments, we became aware of confirming results being published for genes already in progress, our results for mutant alleles of these genes are included in supplemental tables but not presented in summaries of newly discovered lethal mutants.

### Genotyping and Gene Expression Analysis of Mutants in Putative Essential Genes

Based on these criteria, ∼1200 T-DNA lines not previously reported to be homozygous lethal were chosen for study and obtained from the Arabidopsis Biological Resource Center (ABRC; http://abrc.osu.edu/) (Figure 1A). These mutants were grown in soil in controlled environment growth chambers in sets of eight and the plants were genotyped in attempts to identify one or more homozygous plants. For 407 genes, a homozygous plant with normal appearance was found for at least one allele, and no alternative homozygous inviable alleles were discovered (Table S2). These were confirmed as homozygous viable lines by progeny testing, and seeds for these 521 mutants were deposited at ABRC (the stock numbers of the new lines submitted to ABRC are given in Table S2). Two hundred seventy of these homozygous mutants were phenotypically analyzed in the Chloroplast 2010 Project and results for these are available at http://bioinfo.bch.msu.edu/2010_LIMS. As previously reported for putative segregating or homozygous viable T-DNA mutants [19], for 268 mutant alleles of 227 genes, no insertion was found in any plants genotyped (described as ‘all wild type’ in Tables S2 and S3). Complete genotyping information is given in Table S3.

Examination of multiple mutant alleles, including those causing conflicting phenotypes, has great value in reverse genetics projects [19]; therefore this study attempted to screen >1 allele for each gene when available. Whenever possible, mutants were chosen with T-DNA insertions in protein-coding exons near the 5′ end of the gene to maximize the likelihood of obtaining a strong loss of function allele (Figure 1B; scores 1, 2 and 4). Unfortunately, 84 genes on our target list only had available mutant alleles with T-DNA insertions in locations less likely to have strong effects on gene expression (blue text in Table S2). These are lines annotated as having allele strength numbers 7–11, with insertions either in the 5′ UTR, very near the 3′ end, or in an intron near the 3′ end (Figure 1B–C). Analysis of new and stronger alleles would be required to determine whether any of these genes encode essential functions. In other cases, only one of the available mutant alleles met the criteria used to predict a strong allele, and this mutation caused lethality, whereas homozygous T-DNA insertion mutants with allele strength scores 7–11 were found.

**Figure 2:**
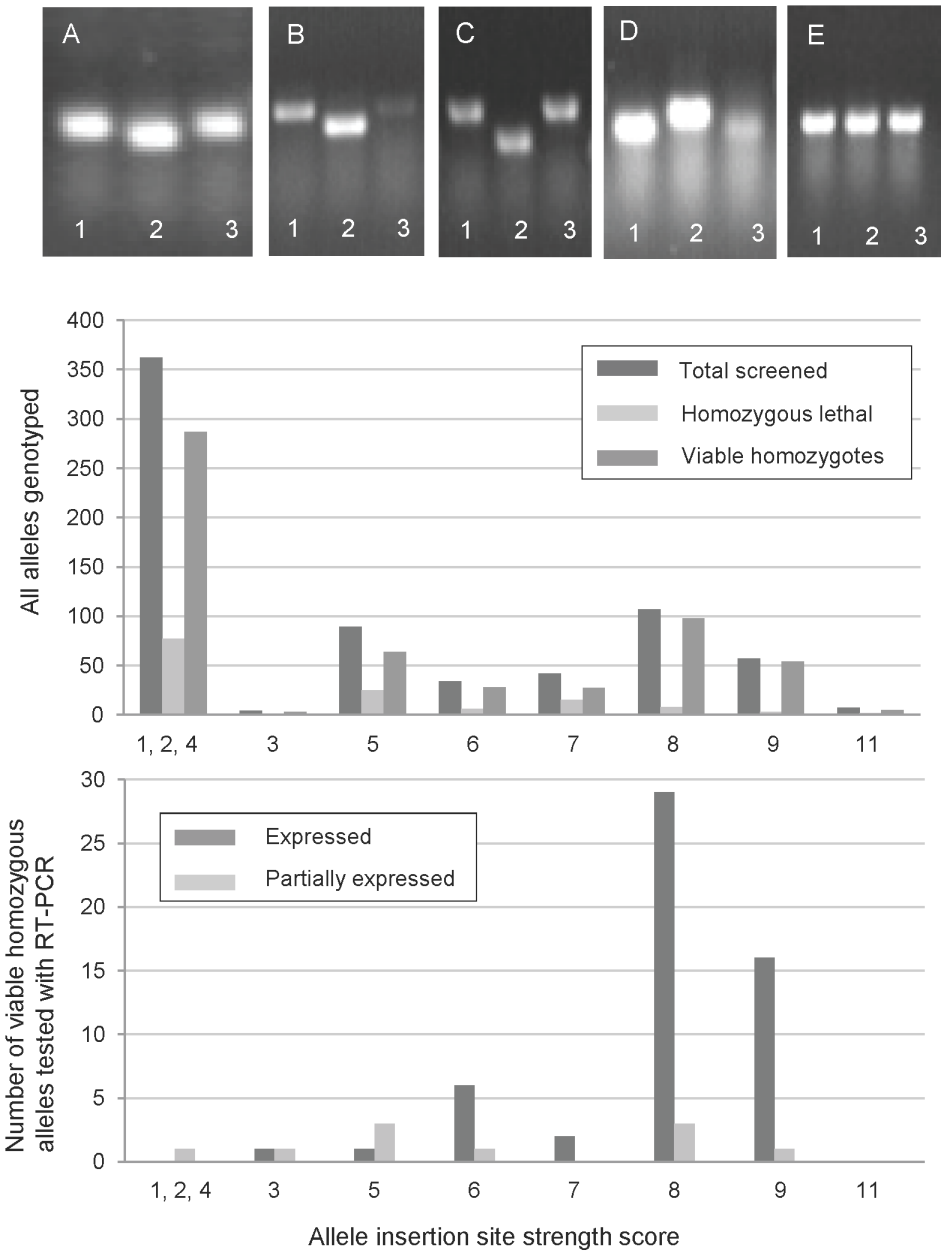
RT-PCR for selected T-DNA mutants, allele strength, and expression. See Figure 1 for explanation of allele strength scores. Top (A – E), RT-PCR for selected T-DNA mutants, A, SALK_039213 (allele score 9), B, SALK_087168 (allele score 5), C, SAIL_373_H09 (allele score 8), D, WiscDsLox477-480L12 (allele score 9), E, SALK_004303 (allele score 3). For each panel, the lanes refer to amplified cDNA from: 1, wild-type Col RNA using gene-specific primers; 2, mutant using ef1α primers; 3, mutant RNA using gene-specific primers. A, C, E: expressed. B, D: partially expressed. Middle, summary of allele strengths for all mutants genotyped, lethal and viable phenotypes. Bottom, RT-PCR results for the tested subset of homozygous viable alleles categorized by allele strength score. No alleles of score 11 were tested.

While we cannot exclude the possibility that, for any individual lethal mutant, inviability is due to a secondary mutation, the large number of genes that show this pattern is consistent with the hypothesis that production of most or all of these proteins is essential for viability. A prediction of this hypothesis is that the viable mutants have leaky alleles. Sixty viable homozygotes were chosen (Figure 2, bottom panel) from this set of predicted leaky mutants and from examples of the 84 mutants for which stronger alleles were not available. These were tested for mRNA accumulation: total RNA was isolated from leaf tissue and analyzed by endpoint RT-PCR using 35 cycles of amplification. All of the homozygous viable mutants tested had at least some detectable mRNA expression (Table S4). 50 of the 60 mutants tested had levels of RT-PCR products indistinguishable from that of wild type (e.g. Figure 2, top, panels A, C and E), and the remaining ten yielded lower RT-PCR product levels than wild type, though mRNA expression was still detected (e.g. Figure 2, top, panels B and D). These results are consistent with the hypothesis that these are essential genes with both strong lethal and weaker viable alleles.

Putative homozygous lethal T-DNA mutant alleles were found for 105 genes not previously reported to be essential (Tables 1 and S1). Additional plants were genotyped to increase confidence that no homozygote could be grown in soil, and to confirm genotypes of plants characterized with the phenotypic assays described below. Numbers of individual plants genotyped were recorded, and these data can be found in Table S1 (column labeled ‘# plants genotyped’). Those that did not produce homozygotes in the initial genotyping tests were assessed to establish whether the mutations caused seed development defects or problems with germination or growth of the seedling. Seed stocks segregating for lethal alleles were sent to the Arabidopsis Biological Resource Center (ABRC; http://www.arabidopsis.org), and the ABRC accession numbers are listed in Table S1, tabs A and B.

**Figure 3:**
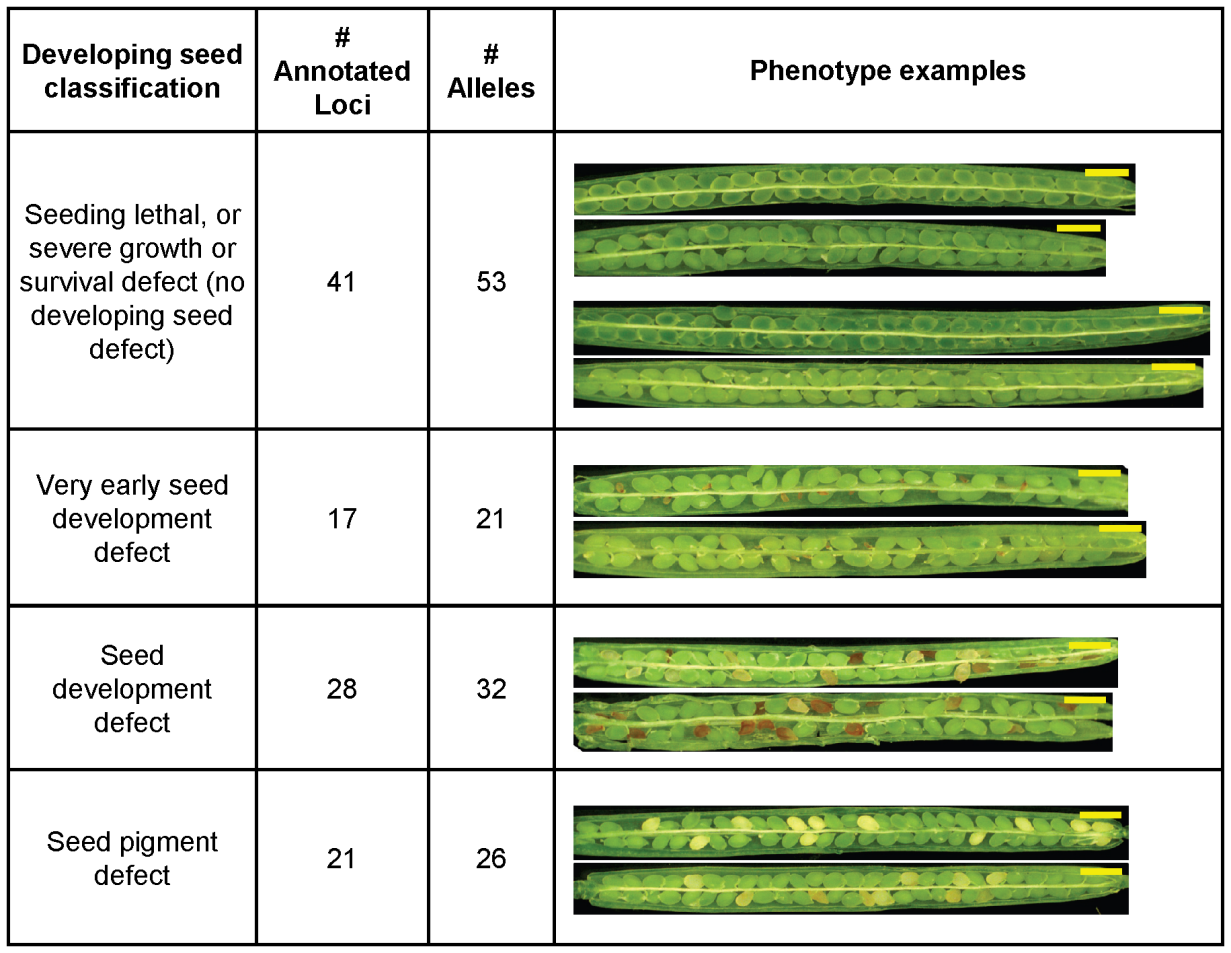
Summary of developing seed phenotypes of homozygous lethal mutants. Summary numbers indicate all mutants from Tables 1 and S1 with no previous identification as homozygous lethal. Morphology classification descriptions are based on our observations, guided by tutorial tools at www.seedgenes.org. Seedling lethal or severe seedling growth or survival defect: seeds appear normal throughout development and at maturity, but seeds do not germinate, or seedlings are not viable. Very early seed development defect: seed development appears arrested very early, sometimes similar to unfertilized ovules. Seed development defect: seed development arrested before full maturity. Seed pigment defect: pigment abnormalities in developing and mature seeds. Phenotype examples, yellow bar = 1 mm. Seedling lethal: SALK_011114 annotated as affecting At2g29630, encoding an enzyme of vitamin B1 (thiamine) biosynthesis; upper image, intermediate stage, lower image, late stage (see text). Bottom two images SAIL_1274_C03 annotated as affecting At4g34090, ‘unknown protein’; upper, intermediate stage, lower, late stage. Very early seed development defect, late stage siliques. Top, SALK_058595, annotated as affecting At2g20690, ‘riboflavin synthase’; bottom, SALK_094586, annotated as affecting At1g03910, ‘unknown protein with cactin-binding domain’. Seed development defect, late stage siliques, top, SALK_014008, annotated as affecting At1g21650, ‘Sec system component’; bottom, SALK_035460, annotated as affecting At5g60750, ‘CAAX amino terminal protease family protein’. Seed pigment defect: late stage siliques, top, FLAG_583E05, annotated as affecting At4g14870, ‘Secretory system component’; bottom, SAIL_810_G07, annotated as affecting At5g01590, ‘unknown protein’.

Classification of a mutant as homozygous seedling or soil lethal can either be strict, excluding any mutant that can produce a homozygous seedling on soil, or more inclusive, encompassing mutants that produce homozygous but severely affected seedlings. Because we observed 17 mutant alleles with homozygous seedlings with severely affected vegetative growth on soil, we chose to adopt the more inclusive definition of lethality, described as ‘severe survival or growth defect’ in Tables 1, S1, and Figure 3.

### Developing Seed Phenotypes of Homozygous Lethal Mutants

Earlier studies showed that the majority of homozygous lethal mutations affect seed development in a way that is readily observed by analysis of seed morphology in the developing fruit [32] (http://www.seedgenes.org/). Because previously-described homozygous lethal seed development mutants were excluded from this study, it was hypothesized that the targeted alleles would be enriched for seedling lethal mutants without developing seed phenotypes, i.e. ‘soil lethal’ mutants. To distinguish mutants with developing seed defects from those with apparently normal seed but seedling germination and survival defects, developing seeds of maturing heterozygous parents were assayed for abnormal morphology and pigmentation. Siliques were split open and photographed *in situ*, the numbers of aborted and abnormally developing seeds were noted, and controlled vocabulary was used to describe the pigmentation of developing seeds (brown, white, light green, yellow, red or orange, or all normal). To increase the probability of observing abnormalities during seed development, developing seed phenotypes were assessed at two stages: intermediate development (wild-type embryos later than torpedo stage but not yet filling the seed coat), and later development (wild-type embryos filling the seed coat, seeds still green, and the silique not yet yellowing). Silique development stages and examples of phenotypes are illustrated in Figure 3. In addition, populations of dry and mature segregating seeds were harvested from heterozygote parents, mixed well to produce a representative population, and photographed. The presence or absence of an apparent segregating mature seed phenotype for the homozygous lethal mutants is noted in Table S1 (see column ‘Abnormal mature seed phenotype’). Silique and mature seed images can be viewed in the Chloroplast 2010 database (http://bioinfo.bch.msu.edu/2010_LIMS), as described below.

Four classes of developing seed phenotypes were observed (Figure 3). Although embryo characterization was beyond the scope of this project, the first three phenotypes are similar to those identified in previous analyses of Arabidopsis embryo and seed development mutants [5]. We describe these as seed development defect mutants, seed pigment defect mutants, and mutants with seedling development defects but no apparent seed morphological changes (seedling lethal). To prevent misclassification at this and other stages of development, a fourth developing seed classification, *very early seed development defect*, is also described (see Figure 3). Mutant seeds that arrest at very early stages of embryo development are often not recovered during bulk seed harvesting and therefore are not observed in dry seed from mature plants. These lines can be confused with rescued seedling lethal lines (see below) or gametophyte lethal lines due to the germination of wild type and heterozygous progeny and lack of homozygous seed. An attempt was made to rule out gametophyte lethality by counting the frequency of the undeveloped seeds from multiple siliques. Mutants were classified as very early developing seed defective when they yielded only heterozygous and wild- type progeny, and if aborted seeds consistently occurred at less than 50% frequency. Despite this, it is possible that some mutants classified as very early seed development defect could be gametophyte lethal. Due to the larger-scale nature of the project, reciprocal crossing experiments necessary to resolve this question or definitively identify gametophyte lethal mutants were not performed. Therefore, putative gametophyte lethal mutants (>50% aborted seeds) are not included in the results presented in Figure 3, and are described as unconfirmed in Table S1, Tab C.

Summary statistics for the full set of homozygous lethal mutants are presented in Figure 3 along with examples of each phenotypic class. Table S5 includes detailed results of all developing seed assays, including analysis of known homozygous lethal controls. This screen allowed us to identify 53 previously uncharacterized mutant lines annotated to 41 loci affected primarily in seedling germination and development, with overall normal seed morphology. Phenotype summary information for 13 loci with mutants of this type confirmed as lethal by two alleles is found in Table 1. Twenty-one genes with a second allele that is hypothesized to be leaky (hypomorphic) based on insertion location and gene expression data (see below) are listed in Table S1.

While these four classifications were useful for characterizing the majority of mutants, a small number of lines defied these descriptions; one example is the putative gametophyte lethal mutants in Table S1. In addition, mutants of gene At1g28140, annotated as encoding a domain of unknown function (DUF) 2301 protein, were conditionally lethal; morphologically normal seed from homozygous parents was viable when freshly harvested, but the seed lost viability after extended storage at 4 degrees C. Arabidopsis mutants that lose viability during storage have been described for genes of a variety of different functions [33], [34].

Recently published curation of loss-of-function phenotypes from the literature [13] showed that only approximately 15% of the mutants classified as essential (110 of 719) were seedling lethal mutants without seed development defects (i.e. pigment defects, embryo defects or gametophyte lethals). We hypothesized that the exclusion of previously discovered seed development mutants [32], [35] would increase the rate of discovery of seedling lethal mutants. Indeed, 38% of the newly annotated genes with mutants summarized in Table S1, Tabs A and B, are defined as seedling lethal. A summary of results for the 25 mutants in 12 genes that have two or more alleles primarily affected in seed germination and seedling development is found in Tables 1 and S1, Tab A. Twenty-one more seedling lethal mutants with only a single allele, and thus requiring further confirmation, were also found (Table S1, Tabs A and C).

### Germination and Growth of Segregating Seed Stocks on Supplemented Media

While far less common than auxotrophs in microbes, Arabidopsis mutants that are not viable or vigorous in soil may be rescued by germinating seed on media containing simple supplements such as sucrose, vitamins, or amino acids [9]–[12], [36]–[39]. Other mutants severely affected in essential pathways such as chloroplast development or chloroplast protein import can germinate, but are albino and typically are only minimally rescued on sucrose-supplemented medium [6], [40]–[43]. To characterize the homozygous lethal mutants for rescue by nutrient supplementation, segregating seed stocks (60–80 seeds) from heterozygous parents were sown on sterile nutrient media with and without addition of sucrose or amino acids. Seed germination was assessed after 7–10 days in the growth chamber, and seedling morphology and pigmentation were noted at approximately 21 days (Table S6). Germination and seedling growth in the presence or absence of 0.5% sucrose were entered into the Chloroplast 2010 database (http://bioinfo.bch.msu.edu/2010_LIMS), along with controlled vocabulary descriptions and photographs of these plates.

**Figure 4:**
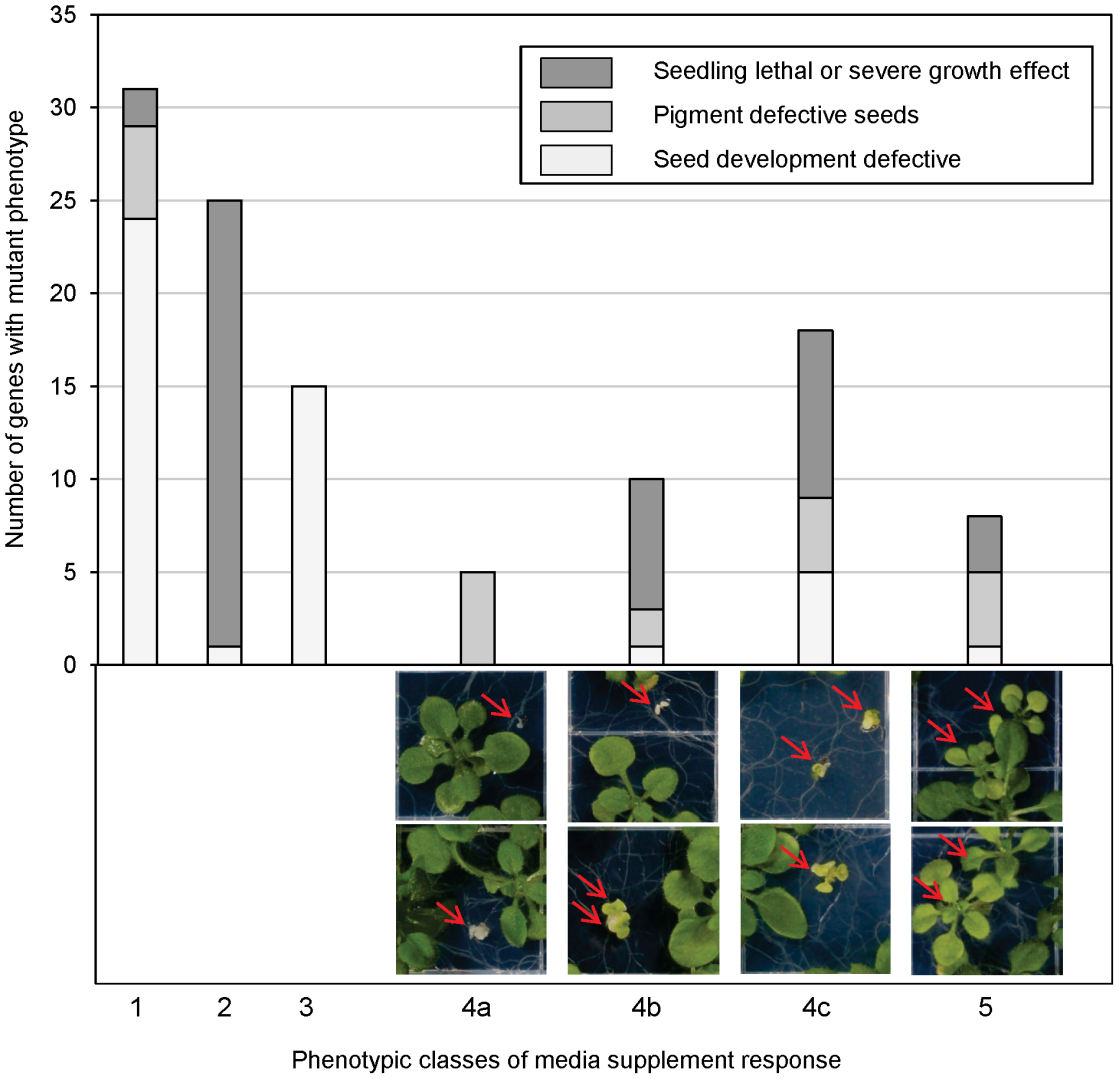
Seedling phenotypes found on supplemented media. Top, phenotype classes (x-axis) refer to portion of segregating seed progeny with phenotype consistently different than wild-type Col sown on the same plate. ‘1’, no rescue on any type of medium (germination ranged from 0 - 89%). ‘2’, germination ≥90% with all seedlings indistinguishable from wild-type Col on all media tested. ‘3’, very early seed development mutants, all seedlings shown or presumed to be heterozygous for the mutation or homozygous wild type. ‘4’, seedling growth with positive response to 0.5% sucrose. ‘4a’, completely albino on all media tested. ‘4b’, albino without 0.5% sucrose supplementation, green with sucrose. ‘4c’, green with or without sucrose supplementation. ‘5’, growth affected by amino acid supplementation. Examples shown: 4a, allele SALK_002470 of At1g63970 encoding 2C-methyl-D-erythritol 2,4-cyclodiphosphate synthase, 4b, allele SALK_056011 of At4g17740 encoding a peptidase S41 family protein, 4c, allele SALK_151530 of At1g50900, encoding *GDC1,* 5, allele SALK_090549 Chloroplast Calvin Cycle sedoheptulose-1,7-bisphosphatase gene At3g55800.

Analysis of seed populations on sterile agar media allowed us to observe and quantify germination and seedling growth. Figure 4 summarizes the classes of mutant seedlings seen, with examples of the phenotypes observed. Phenotype examples are not shown for classes 1 and 3 because there are no germinated putative homozygous seedlings, or for class 2 because all seedlings are indistinguishable from wild-type Col. Mutants with severe seed development defects comprise the majority of those with a low germination phenotype (class 1). Not surprisingly, many seedling lethal or severe survival or growth mutants grew better on sterile agar basal medium (Figure 4, class 2), than on soil mix. On sterile media these seeds germinated at high frequency and the resultant seedlings resembled wild-type Col (Tables 1, S6). Class 3 mutants are classified separately; in these cases homozygous mutant seeds aborted very early in development and the only macroscopic seeds produced were homozygous wild type and heterozygotes. This caused Class 3 seed stocks to have a high frequency of germination and fully normal seedlings (Table S6). These characteristics could be incorrectly interpreted as a rescued seedling lethal phenotype. However, these mutants were distinguished by their very early developing seed defect phenotype (Figure 3).

Sucrose and amino acid supplementation revealed a variety of types of mutants and degrees of responses to supplementation. Examples for sucrose supplementation and responses to amino acids are shown in Figures S1 and S2, respectively. Class 4a mutants are albino seedlings without true leaves in the absence of sucrose, and addition of the carbon source to the medium caused these lines to produce one or more pairs of true leaves (see also Figure S1A–S1D), although the seedlings remained albino. In contrast, class 4b mutants produce albino seedlings without true leaves in the absence of sucrose, but sucrose supplementation results in green seedlings with true leaves. No seedling lethal mutants that could be rescued by sucrose had completely albino seedlings on any sterile medium tested; instead, rescued seedlings for seedling lethal mutants were only found for green or conditionally green classes 4b, 4c and 5 in Figure 4, with the majority found in class 4b. A small number of mutants with an increased response to additional sucrose (2% vs. 0.5%) are indicated in Tables S1 and S6 as class 4d. Seeds of class 4a and 4d mutants are all pigment defective (Figures 3 and 4). Class 4c mutants are green with or without sucrose supplementation, but show improved growth on sucrose (also see examples in Figure S1E and S2F). This type of seedling response on media was the most varied; examples of mutants with seed development, seed pigment, and seedling lethal mutants fell into this class.

As predicted from examples in the literature, annotations for sucrose responsive genes vary widely. These include mutants in already characterized loci, which were not previously tested for response to medium supplementation. Examples include the plastidic non-mevalonate isoprenoid biosynthetic enzyme 2C-methyl-D-erythritol 2,4-cyclodiphosphate synthase (At1g63970; [44]), impaired in synthesis of photoprotective carotenoids, and *GDC1* (At1g50900; also called *LTD*), which plays a role in thylakoid biogenesis [45]. There also are mutants defective in previously uncharacterized genes in this category; for example, mutants of At1g09820, a pentatricopeptide (PPR) repeat-containing protein, and those defective in the putative ‘transketolase’ gene At3g60750.

Relatively few mutants showed responses to the mixture of amino acids employed in this study, consistent with the paucity of previously characterized amino acid-requiring mutants. Mutants of the PPR domain-containing protein At1g09820 (Fig. S2A, compare top to bottom panels) and At3g55800, defective in the Calvin-Benson Cycle enzyme sedoheptulose-1,7-bisphosphatase (Figure S2B), were notable for responses to amino acid supplementation. Because of the broad roles of PPR proteins in organelle gene expression, and the central importance of the Calvin-Benson Cycle, it is not clear whether these proteins directly influence amino acid homeostasis.

Two other types of responses were observed. Figures S2C and S2D show examples of mutants whose growth was positively impacted by amino acid supplementation, but to a more modest extent (in each case compare left image, without amino acids, to right image, with supplementation). In contrast, one of the most unusual responses to supplementation was observed for survival and growth defective mutants of At3g12290, annotated as an amino acid dehydrogenase family protein. While small seedlings were partially rescued on basal medium, growth was *inhibited* on both sucrose and amino acids (Figure S2E right 3 panels). Imbalance between specific amino acids is a potential mechanism for reduced vigor in supplemented medium; a well-documented example of this phenomenon is seen when plants are grown on a mixture of Lys+Thr, causing methionine auxotrophy due to inhibition of aspartate kinase [46]. Alternatively, one or more supplement breakdown products could be influencing growth of the mutants. Tests with individual amino acids or mixtures of metabolically related combinations would be needed to begin to understand the mechanism of the phenotypic responses to amino acid supplementation.

### Screen for Rescue Under Non-photorespiratory Conditions

While decades of study have led to a detailed understanding of photorespiration, transporters and other proteins that influence the pathway continue to be discovered [47]–[49]. To identify previously unreported mutants defective in photorespiration or otherwise responsive to CO_2_ enrichment, 12–32 seeds from segregating populations derived from individual heterozygous parents segregating for lethal progeny were sown in soil and grown in air with and without 0.3% CO_2_ enrichment. Col wild type and *shm1-1* [50], a CO_2_-responsive photorespiratory mutant defective in mitochondrial serine hydroxymethyltransferase, were grown in each flat as controls. Detailed results of the screen are presented in Tables 2 and S7, and Figure S3 shows examples of controls and mutants from this study. The percentage of seeds that germinated after 7–10 days growth was noted, seedling survival and any abnormal appearance was assessed again after three weeks of growth, and seedling size, pigmentation, and the morphology was summarized (Tables 2 and S7, columns ‘abnormal morphology air’ and ‘abnormal morphology high CO_2_’). Mutants that did not respond to high CO_2_ were not screened further (Table S7, Tab B).

**Table 2:** Mutants responsive to non-photorespiratory conditions.

Annotated Locus	Allele	Allele score^a^	Phenotype summary	n = b		% germination		% survived^c^		% abnormal^d^		Abnormal morphology at three weeks	
				air	high CO_2_	air	high CO_2_	air	high CO_2_	air	high CO_2_	air	high CO_2_
At1g05385	SALK_093605^e^	2	germination, survival, morphology	4	4	87.4	93.9	69.8	86.1	21	15.6	very small/tiny light green	very small/tiny light green
At1g23400	SALK_008478^f^	7	germination	2	4	71.5	87.8	55.9	74.0	0	0	none	none
At2g21380	FLAG_299H06^g^	5	survival, morphology	5	7	93.9	98.4	79.8	96.1	17.2	15	very small, lighter green, necrotic	small, lighter green, necrotic
At3g12590	SALK_044513	4	germination, morphology	6	7	91.7	87.2	82.6	80.8	6.8	5	tiny dark green	small dark green
	SALK_129651	5	no difference	6	8	95.5	95.6	93.8	92.7	13.4	10.3	very small dark green	very small dark green
At3g15190^h^	SALK_132553	9	morphology	2	2	100.0	100.0	91.7	100.0	16.7	16.7	tiny light green	very small light green
	SALK_094710	7	no difference	1	2	75.0	70.8	75.0	70.8	ND	0	ND	none
At3g17930	SALK_088638	4	survival	3	8	92.9	95.7	73.2	85.3	0	11.2	none	tiny
	FLAG_202E10	3 or 8	no difference	4	4	95.8	86.4	83.3	86.4	0	0	none	none
At4g14605^i^	SAIL_425_E03	2	slight difference, morphology	3	5	100.0	97.5	96.4	95.0	21.4	20	small light green necrotic	small light green necrotic
	SALK_097243	4	germination, morphology	3	5	91.1	96.3	82.1	90.0	23.2	18.75	small or tiny light green necrotic	small light green necrotic
At4g17620	SALK_032903	5	survival	3	5	83.9	88.8	69.6	82.5	3.5	13.8	very small light green, abnormal morphology	very small light green, abnormal morphology
	SALK_103157	1	survival	3	4	80.4	80.6	75.0	79.2	7.1	16.7	very small light green, abnormal morphology	very small light green, abnormal morphology
At1g80380^j^	SALK_073728	8	control; morphology	2	2	85.6	90.0	73.3	80.6	100	0	small	none
At5g04140^k^	SALK_011035	7	control; survival	2	2	85.2	96.3	66.7	96.3	100	100	very small yellow	light green normal size

High CO_2_ is 0.3%, air is ambient atmosphere. Mutants presented are those with numbers of seedlings with germination, survival, or morphology differences significant at p = 0.05 or less (Student’s T-test). Homozygous genotypes for seedlings with abnormal morphologies were confirmed by PCR except SALK_008478.

a see Figure 1.

b n = , number of discrete growouts conducted in indicated conditions.

c percentage of seedlings that survived to three weeks old in indicated conditions.

d percentage of seeds sown that yielded a seedling with described abnormal appearance.

e See [52] for additional characterized alleles that could be screened.

f Germinated albino seedlings did not survive to 3 weeks; stronger alleles are embryo lethal [53].

g Additional alleles could be screened to confirm result.

h See also [86].

i [81].

j [87].

k [48].

Mutants with seedlings rescued by CO_2_ were grown again in air and high CO_2_ for further comparison (Table 2, and Table S7, Tab ‘A’). All mutants that responded were retested 2 or more times (Tables 2 and S7, columns ‘air n = ’ and ‘high CO_2_ n = ’) to assess the reproducibility of the CO_2_ response. Numbers of germinated and abnormal seedlings for the two conditions were compared and responses were considered significant at p≤0.05 (Student’s *t*-test). The homozygosity of surviving putative rescued mutant plants was validated by genotyping with allele-specific assays. Table 2 summarizes those homozygous mutants with reproducible germination, growth, or seedling survival differences in unsupplemented air vs. 0.3% CO_2,_ (Table 2, see column ‘phenotype summary’). The remainder of the mutants did not display consistent differences when directly compared (Table S7, Tabs A and B).

Mutants with alleles in eight genes not previously reported to influence growth under CO_2_ enrichment were identified as having improved growth or survival compared with ambient air (Table 2). Mutant SALK_088638, (At3g17930), annotated to the thylakoid membrane protein involved in the accumulation of the cytochrome b6/f complex and recently described in [51], showed an absolute requirement for CO_2_ supplementation for germination and growth on soil. The others showed varying degrees of positive response, including variations in germination, frequency of survival of homozygotes, and changes in morphology, typically displaying a larger seedling size. The annotation of these mutant lines varies widely, including unknown proteins (Figure S2), and several with putative functions in different steps in transcription or translation (e.g. At3g15190, a putative chloroplast 30S ribosomal protein). Genes with mutants previously reported to have other mutant phenotypes are included in the summary table (Table 2). These include At1g05385, a homologue of the PSII protein PSB27, reported to function in C-terminal processing of the PSII D1 protein [52], and SALK_008478, containing an insertion in the last intron of At1g23400, previously described for the stronger embryo lethal allele *caf2-1* (SALK_049304 [53]). More detailed studies are required to determine whether the improved growth of some mutants in 0.3% CO_2_ is due to a direct impact on photorespiration or more or less direct CO_2_ fertilization effects [54], [55].

### Mutants Affected by Growth in Non-photorespiratory Conditions that Display Alterations in Chlorophyll Fluorescence

A variety of published studies employed measurements of PSII maximum quantum efficiency (Fv/Fm) and non-photochemical quenching (NPQ) to investigate links between photorespiration and photoprotection [48], [56]–[60]. Increased non-photochemical quenching or decreased Fv/Fm after transfer from air enriched with CO_2_ to ambient CO_2_ conditions was reported for a variety of Arabidopsis photorespiratory mutants. These include lines defective in the enzymes hydroxypyruvate reductase, 2-phosphoglycolate phosphatase, chloroplast ferredoxin-dependent glutamate synthase (Fd-GOGAT), mitochondrial serine hydroxymethyltransferase and glycerate kinase (GLYK), as well as the chloroplast envelope glutamate/malate transporter (DiT2), [56], [59], [60]. This pleiotropy is not restricted to Arabidopsis photorespiratory mutants: decreased Fv/Fm was seen in rice and tobacco glycolate oxidase mutants measured in air [58], [61], and reductions in Fv/Fm indicating increased photoinhibition in high light were reported in a glutamine synthetase mutant of tobacco [57].

**Figure 5:**
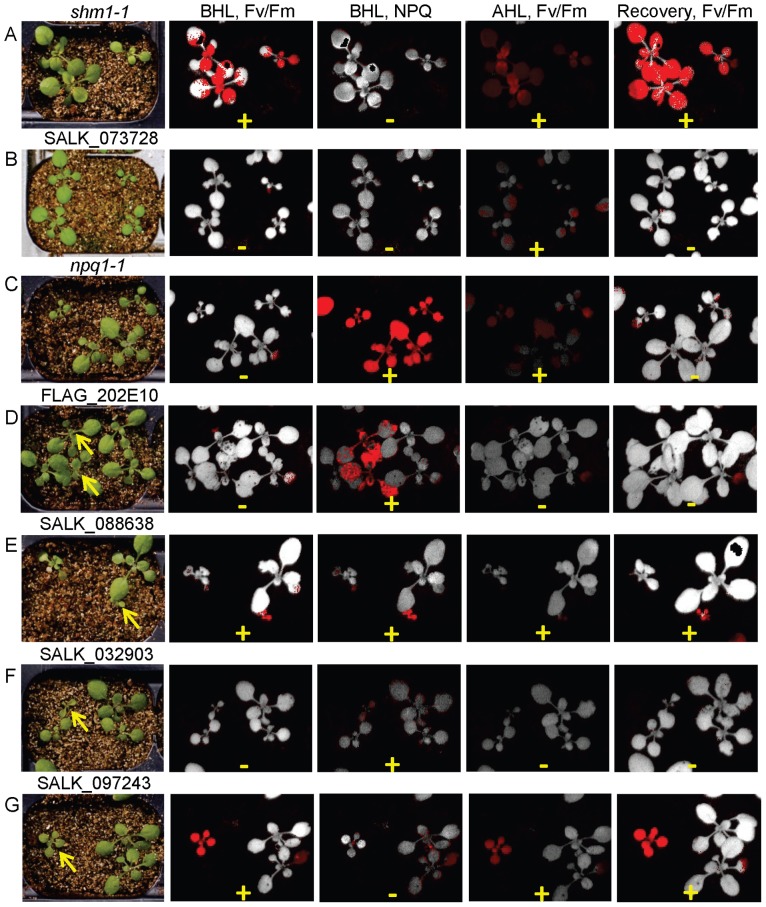
Chlorophyll fluorescence false color images for mutants with altered growth in 0.3% CO_2_. Plants shown at left photographed after growth on enriched CO_2_ and before analysis. The meanings of the column headings and cutoffs for ‘+’ and ‘−’ are as in Table 3. Arrows indicate homozygous mutant plants. A, *shm1-1*. B, SALK_073728, an allele of glycerate kinase. C, *npq1-1*. D, allele FLAG_202E10 of At3g17930. E, allele SALK_088638 of At3g17930. F, allele SALK_032903 of At4g17620. G, allele SALK_097243 of At4g14605.

Chlorophyll fluorescence parameters were measured to determine whether the CO_2_-responsive mutants we identified also have altered photosynthetic physiology. Two week old mutants were transferred from 0.3% CO_2_ in the light to unsupplemented air in the dark for 20 minutes prior to measuring Fv/Fm and NPQ (Table 3, ‘Fv/Fm BHL’ and ‘NPQ BHL’). Response of the mutants to a three-hour photoinhibitory high light period (1,500–1,700 µmol photons/m2/s) in air was also measured (Table 3, ‘Fv/Fm AHL’). Finally, the ability of the mutants to recover from high light stress in unsupplemented air was measured after a two-day recovery period in the original 0.3% CO_2_ growth conditions (Table 3, ‘Fv/Fm Recovery’). Mutants of four of the eight genes with enriched CO_2_-responsive mutants (Table 2) also showed alterations in chlorophyll fluorescence parameters (Table 3). Figure 5 shows examples of false color image results for one or more alleles of each gene of these four genes.

**Table 3:** Chlorophyll fluorescence measurements for mutants affected by growth in nonphotorespiratory conditions.

Gene	Allele (score)	Fv/Fm BHL	NPQ (BHL)	Fv/Fm AHL	Fv/Fm Recovery	Growth or survival affected by non-photorespiratory conditions?
At3g17930 [51]	SALK_088638 (4)	+	+	+	+	yes
	FLAG_202E10 (3)	−	+	−	−	no
At4g17620	SALK_032903 (5)	−	+	−	−	yes
	SALK_103157 (1)	+	+	−	+	yes
At4g14605 [81]	SALK_097243 (4)	+	−	+	+	yes
	SAIL_425_E03 (2)	+	−	+	+	slight
At1g05385 [52]	SALK_093605 (2)	+	−	+	+	yes
Photorespiration mutant controls:						
At1g80380 [87]	SALK_073728	−	−	+	−	yes
At4g37930 [48]	*shm1-1*	+	−	+	+	yes
Controls, chlorophyll fluorescence measurements:						
At1g08550 [70]	*npq1-2*	−	+	+	−	ND
At3g56040 [71]	SALK_020654	+	+	+	−	ND
At5g13050 [62]	5-FCL	−	+/−	+	−	ND

Controls are described further in text. Fv/Fm, +, measurement indicating reduced Fv/Fm, −, measurement indicating no difference compared to wild type Col. NPQ, +, measurement indicating increased NPQ, −, measurement indicating no difference compared to wild type Col. BHL, before high light treatment, AHL, after high light treatment, Recovery, after 2 days return to original growth conditions. ND, direct air vs. high CO_2_ comparisons were not done for these mutant controls.

Previously reported alterations in Fv/Fm or NPQ for photorespiration mutants were measured under a variety of conditions [56], [57], [59], [60]. For this reason, characterized photorespiration mutants were included in the assays to aid in the interpretation of the results. In fact, glycerate kinase mutant SALK_073728 had reduced Fv/Fm after high light treatment and *shm1-1* had reduced Fv/Fm at all three conditions measured [56]. Interestingly, although it was not previously reported to have photosynthetic defects, the photorespiratory 5-FCL (5-CHO-tetrahydrofolate cycloligase [62]) mutant also had decreased Fv/Fm after high light treatment under the high CO_2_ growth conditions of this study (Table 3).

Photosynthetic defects were observed for several of the CO_2_-responsive alleles discovered in this study. SALK_088638, the stronger of the two At3g17930 alleles, had high CO_2_-dependent germination and growth (Table 2). These very small CO_2_-rescued mutants were affected in all four photosynthetic parameters tested (Figure 5E). In addition, the normal morphology hypomorphic mutant of this gene, FLAG_202E10, had increased NPQ (Figure 5D). It is possible that the less severely affected mutant allele (compare Figure 5D and 5E) was not affected by the switch from high CO_2_ to air, and also that the increased NPQ for mutants of this gene is not correlated with dependence on high CO_2_. Two mutants of At4g14605, annotated as a plastid-localized mitochondrial transcription termination factor family protein [63], showed chlorophyll fluorescence alterations similar to *shm1-1*, with decreased Fv/Fm in all three measurements, but no alteration in NPQ. The same phenotype was also observed for SALK_093605, a mutant of PSB27 homolog At1g05385. Finally, both mutants of At4g17620, a glycine-rich protein of unknown function had altered Fv/Fm and NPQ before high light treatment. Because these mutants did not show photosynthetic alterations after the three- hour high light treatment in air (Table 3, Fv/Fm AHL), the link to the high CO_2_ response is not clear.

These results do not appear to be due to a general stress effect because two other growth defective mutants and lines that responded to CO_2_ enrichment showed no chlorophyll fluorescence alteration (Tables S1 and S7). For example, SALK_044513 (see also Figure S3), a stronger allele of unannotated gene At3g12590, and FLAG_299H06, a single mutant of At2g21380, a kinetin family protein, both responded positively to non-photorespiratory conditions but did not have alterations in Fv/Fm or NPQ. Mutant SALK_008478 [32] could not be analyzed because the albino homozygous seedlings that germinated under high CO_2_ conditions did not survive long enough to assay for chlorophyll fluorescence.

### Annotation of Genes of Unknown Function

A major goal of the broader Chloroplast 2010 phenomics project, and this screen for lethal mutants in particular, was to provide phenotypic annotation for genes with little or no published functional annotation. This objective was realized in that approximately a third of the genes with confirmed or putative lethal mutant phenotypes are currently without functional annotation in TAIR. Pentatricopeptide (PPR) repeat-containing proteins with unknown functions were a particularly rich source of genes with lethal mutant phenotypes, consistent with previous published studies [28], [29], [31]. Out of 40 PPR proteins tested, 10 were identified as homozygous lethal with seed development or pigment defective phenotypes. Of this group, only mutants of At1g09820 could be rescued on sucrose- or amino acid-supplemented media (Table S6, Figure S2A). At2g28390, a SAND family protein with no functional annotation, was the only gene that had mutants with mature seeds of normal appearance, but low germination on sucrose or non-sucrose containing media. Little information on gene function is available, except for the presence of a conserved MON1-like domain thought to be associated with membrane fusion and metabolite trafficking in yeast and other non-plant eukaryotes [64]. The gene is constitutively and widely expressed and is sometimes used as an expression reference gene; hence the many associated references at TAIR. Given the broad expression pattern, it is notable that the mutation impacts both seed germination and seedling growth rather than seed development. One gene of unknown function has seedling lethal mutants with some of the most pleiotropic phenotypes found in the study: At4g17620, encoding a glycine-rich protein with a RA1-like domain. Mutants of this gene respond to sucrose supplementation, non-photorespiratory conditions, and have increased nonphotochemical quenching, indicating a direct or indirect involvement in photosynthesis (Tables 1, 3). More detailed analysis of these and other essential ‘genes of unknown function’ should provide insights to researchers with interests in specific protein families or biological functions.

While testing multiple alleles may confirm a homozygous lethal phenotype, one or more additional non-homozygous lethal mutants are required to discover phenotypes beyond lethality. We discovered eight such genes with two lethal alleles and an additional viable homozygous allele (Table S1). For example, mutants of At1g71720, encoding an S1 RNA-binding domain-containing protein with insertions in the second or seventh exons, have seedling lethal phenotypes (SAIL_162_G11 and SALK_107226). In contrast, an insertion 102 bp 5′ from the first annotated exon of this gene (SALK_127604) does not affect gene expression and is viable (Tables 1, S1, S4). The theme of a single viable weak allele and one homozygous lethal strong allele was observed for 29 genes (Table S1). Although the lack of two alleles with similar phenotypes (for example, two lethal mutations or two leaky alleles) prevent us from definitive conclusions regarding functions of these genes, comparing phenotypes associated with stronger amorphic mutations and hypomorphic leaky lesions is sometimes instructive. For example, At3g15190, annotated as a putative chloroplast 30S ribosomal protein S20, has a developing seed defect phenotype when the insert is in the second intron (SALK_094710, Table S1), but a seed pigment and severe growth defect when the insert is 83 base pairs upstream from the first exon (SALK_132553). Analysis of a leaky mutant of some genes allowed observation of responses to sucrose and amino acid supplementation as well as 0.3% CO_2_ (Tables 2 and S1). For example, CO_2_-responsive mutant SALK_008478 (Table 2) is a weaker allele of embryo lethal *caf1-2* [53]. In contrast, a small number of anomalous mutants had lethal phenotypes regardless of the T-DNA insertion location. For instance, mutants of genes At5g42390 and At2g41950 had seed development defects whether the insertion was in the 5′ UTR (scores 8 or 9, Figure 1) or in an exon (Table S1); no homozygous seedlings could germinate under any tested condition. This was also true for seedling lethal mutants of At2g28390 with insertions in either the last intron or the first exon (Table 1, S1). Other approaches, perhaps including analysis of new mutations, will have to be pursued to discover functions for these genes.

## Conclusion

Despite remarkable breakthroughs in genome science technologies during the past decade, the discovery of function for each of the tens of thousands of genes in a typical multicellular eukaryote continues to present a daunting challenge. This problem is especially acute for proteins of unknown function lacking well-characterized homologues, because it is difficult to choose assays likely to reveal a mutant phenotype. Screening for lethal mutants is an exception to this general theme: seed development defects and seedling lethality can be assessed in a standardized, though time consuming, manner. Even when these phenotypes are discovered, obtaining more phenotypic annotation for homozygous lethal mutants requires detailed analysis (for example see [65]). In this study we attempted to overcome these challenges in two ways: attempting to rescue seedlings of lethal mutants by supplementation with nutrients and CO_2_, as well as through analysis of leakier alleles of essential genes.

Availability of EMS mutants and one or more insertion mutant in the majority of Arabidopsis genes [66] previously led to documentation of >700 genes with lethal mutants [13]. In the current study, systematic screening of ∼1200 mutants annotated as being defective in ∼600 Arabidopsis nuclear genes enriched for plastid targeted protein-coding sequences led to the identification of confirmed or putative lethal mutants annotated as defective for 105 genes (18% of the genes tested), increasing the number of validated or potential essential genes annotated in the Arabidopsis genome [13]. This success rate is high enough that identification of the vast majority of the remaining essential plastid functions in Arabidopsis could be accomplished with a relatively modest investment of resources. Such a comprehensive catalogue of essential plant genes would be a valuable resource for development of new crop protection strategies as well as increasing our fundamental understanding of plastid physiology.

While Arabidopsis has sequenced insertion mutant resources unparalleled in plants, continued improvement in these and other mutant assets is required to enable identification of functions for every Arabidopsis gene. It was recently reported that ∼12% of Arabidopsis genes do not have insertion lines available in previously generated collections [66]; consistent with this general statistic, >600 of the >5300 genes on the Chloroplast 2010 target list currently do not have any confirmed homozygous alleles available. The situation is especially acute for specific classes of proteins that were not previously targets of intensive study. For example, no alleles are available for four of eight Domain of Unknown Function (DUF) 241-containing proteins (there are no alleles for At2g17080, At4g35200, At4g35210, At4g35710; mutants are available for At2g17070, At3g51410, At4g35660 and At4g35720).

Two other lines of evidence suggest that the lethal mutants discovered in this study may be an under-representation of the actual number of essential genes on the target list. First, we were unable to obtain complete loss of function (amorphic) alleles for 84 genes based upon semi-quantitative RT-PCR and position of the T-DNA, consistent with the hypothesis that many viable mutants could be leaky alleles of essential genes. Second, it was not possible to identify either homozygous mutant or segregating populations containing the annotated T-DNA insertion in mutants for another 66 genes (genotype result ‘all wild type’ in Table S3).

In contrast to microorganisms, identification of plant lethal mutants rescued by varying the growth conditions is labor intensive and infrequently attempted [8], [9], [12], [36], [37]. In the current study, populations segregating for seed and seedling lethal mutants were screened for rescue by sucrose, a mixture containing amino acids, or CO_2_ enrichment. In addition to discovering hitherto uncharacterized lines that respond to one or more of these treatments, we also found examples of previously described lethal mutants whose growth was improved by supplementation. For example, mutants of the 2-C-methyl-D-erythritol 2,4-cyclodiphosphate synthase gene At1g63970 [67], [68] were previously shown to be chlorotic on medium containing 2–3% sucrose. Our studies show that these lines grow even more poorly without added carbon source. We also found that a mutant of the previously reported *GDC1* gene At1g50900 [45] responds to sucrose and amino acid supplementation.

**Figure 6:**
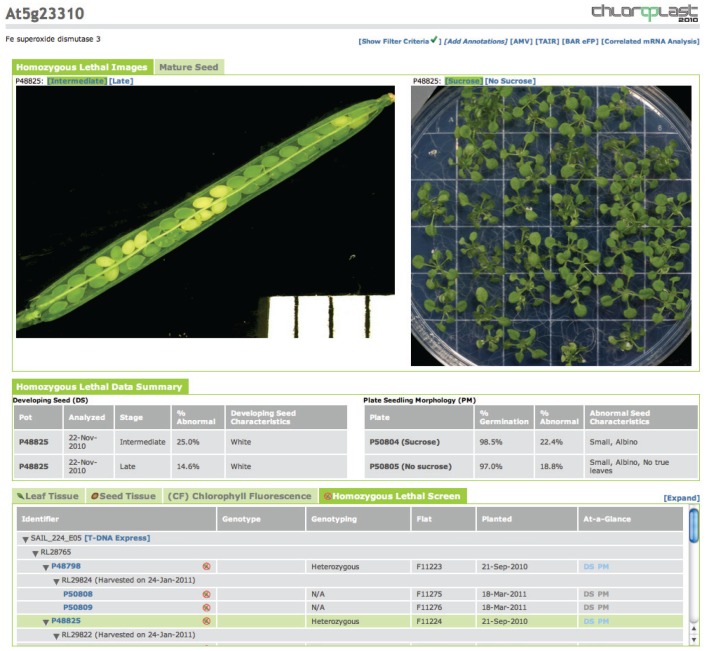
View of homozygous lethal mutant results in Chloroplast 2010 database. This shows an example of the user interface at: http://bioinfo.bch.msu.edu/2010_LIMS. Results for specific mutants or loci can be found through ‘Search by Query Term’ for AGI (Arabidopsis Genome Initiative) number or allele names. Alternatively, choosing ‘Search Lethals Screen’ returns a list of all genes with assay results for developing seed or growth on supplemented media. Clicking on the AGI gene identifier presents a ‘gene page’, which is divided into three panels: images are displayed at the top, controlled vocabulary describing the image results are presented in a middle panel, and a comprehensive summary of the pedigree and genotype and phenotype assay results for all tested mutants of the gene is found in the bottom panel. Genes with mutants screened in this study have an additional tab in the bottom panel, ‘Homozygous Lethal Screen.’ The color of the ‘At-a-Glance’ icons for developing seed (DS) assays and morphology of seedlings grown on nutrient media plates (PM) indicates the presence (blue) or absence (gray) of assay results for each lineage. When an individual having assay data is selected, two types of data specific to the homozygous lethal screen can be viewed in the middle and top panels. Developing seed images appear on the top left. The silique stage (late or intermediate, see information on developing seed phenotypes, above) and percentage of developing seeds with abnormal pigmentation (brown, white, light green, yellow, red or orange, or all normal) are presented in the middle left as controlled vocabulary. A millimeter ruler is visible in each silique image to facilitate comparisons between images. Results from the screen for seedling growth on basal medium or medium supplemented with 0.5% sucrose are shown on the right, with Col wild type control seedlings visible in the top left sector.

This work was part of a larger phenomics project (http://www.plastid.msu.edu) that sought functional annotation for several thousand genes, and it continues the philosophy of making large sets of data available in expectation that experts in the scientific community will make discoveries beyond those that we report here. For example, images and assay results from this study are accessible online at the Chloroplast 2010 database (http://bioinfo.bch.msu.edu/2010_LIMS and Figure 6). General query tool instructions and a database overview can be found by accessing ‘Help’ at this site, and in a recent publication [23]. In addition, comprehensive data from each step in the project are included in the supplemental tables, whether or not the mutants were identified as newly discovered essential genes. We also include information even when the connection between genotype and phenotype is not conclusive: for example when existing annotation of the T-DNA insertion site is not definitively supported by genotyping data or when a second allele was not available or did not cause a phenotype that agreed with the first mutant. As with any high throughput project, judicious use of the data from this project should be useful to plant scientists interested in a wide variety of biological processes.

## Materials and Methods

### Plant Growth

For initial genotyping, plants sown from segregating seed stocks were grown in soil in sets of four plants per 2.5 square inch pot, eight plants per mutant total, in controlled growth chambers at 21 degrees C, 16 hours cool-white fluorescent light, 8 hours dark photoperiod. All other details of the plant growth protocol were as described for the Chloroplast 2010 phenotyping project [20].

### Genotyping

For each mutant allele, leaf samples were archived using FTA cards (GE Healthcare Life Sciences, Piscataway, NJ). Samples from at least eight plants were tested for genotype by PCR as described [19], [20]; primers employed are described in Supplemental Table S3. When no homozygote was found in eight or fewer surviving plants, we considered the possibility that homozygote seeds might be underrepresented in the segregating seed or have lost viability during storage as seen for tocopherol and amino acid metabolism mutants [33], [34]. Heterozygotes were self-pollinated to propagate additional fresh seed, and these progeny plants were genotyped. Homozygotes that were recovered were grown to maturity, seed was harvested, and progeny from the harvested seed were grown and genotyped as above for confirmation.

### Developing Seed Assay

Developing seeds were viewed and photographed using a Leica MZ12.5 high-performance stereomicroscope (Leica Microsystems, Wetzlar, Germany) with 1× objective lens and SPOT Insight Color 3.2.0 digital camera (Diagnostic Instruments, Sterling Heights, MI).

### Rescue by Medium Supplementation

To assess response to sucrose or amino acid supplementation, harvested segregating seed stocks were mixed well and aliquots of 60–80 seeds were surface-sterilized by washing in 70% ethanol for 1 minute and 10% bleach for 20 minutes. Sterilized seeds were sown on up to five different media in 25×150 mm Petri plates. The basal medium was 0.5X Murashige and Skoog Basal Medium with Gamborg’s B5 vitamins (containing myo-inositol, nicotinic acid, pyridoxine hydrochloride, thiamine hydrochloride) (Sigma-Aldrich, St. Louis, MO), and 7.5 g/L agar (Bacto agar, BD Corporation, Franklin Lakes, NJ). Sucrose was added as indicated before autoclaving, and the amino acid supplement mixture (final concentration 0.1X) was made from two sterile Minimum Essential Medium (MEM) amino acid mixtures (Sigma-Aldrich, St. Louis, MO), added after the autoclaved medium was cooled to 60 degrees C. Final concentration of amino acids was 60 µM arginine, 10 µM asparagine, 10 µM aspartic acid, 10 µM glycine, 10 µM cystine, 10 µM proline, 10 µM alanine, 10 µM glutamic acid, 20 µM histidine, 40 µM isoleucine, 40 µM leucine, 40 µM lysine, 10 µM methionine, 20 µM phenylalanine, 10 µM serine, 40 µM threonine, 5 µM tryptophan, 20 µM tyrosine, 40 µM valine. Plates were sealed with porous paper tape (3M “Micropore” Tape, 3M, St. Paul, MN), stratified at 4°C for 2–4 days to increase germination efficiency, and then moved to a controlled environment growth room. Light levels, temperature, and photoperiod were the same as for growth in soil [20]. Seedlings were compared to Col wild type (ABRC stock CS60000) grown in the same position on every assay plate. For consistency, images of seedlings growing in media were processed in Adobe Photoshop CS4 with an 18% gray card. Because seedling growth can be inhibited at high concentrations of individual amino acids or in combinations, supplemented media were tested for efficacy by growing tryptophan synthase auxotrophs *trp3-1* and *trp2-1* [36] to find the minimum concentration of amino acid mixture that demonstrated growth enhancement of the mutants without causing growth inhibition of wild-type seedlings (See Figure S4 for response of these mutants to medium supplementation). Individual seedlings were genotyped in cases where it was difficult to interpret phenotyping results. Variability was observed in the growth of seedlings of homogeneous genotype on various media, and this should be considered when interpreting controlled vocabulary assessments of seedling morphology in Table S6 or in the Chloroplast 2010 database. ‘Arrested’ seedlings were especially problematic; these did not progress in growth beyond germination and cotyledon expansion. Seedlings of this type are described in this study as ‘small, light green, with no true leaves.’ On medium not containing sucrose, seedling arrest was quite variable in wild-type Col, occurring at a rate of 0–12%. While some homozygous mutants have a phenotype of ‘small light green seedlings with no true leaves’, which is strongly associated with the mutation and not simply due to the absence of sucrose supplementation, genotyping of others showed that many seedlings with an arrested appearance were heterozygous or wild type. In some batches of mutant seeds, the frequency of arrest ranged from similar to wild type to as much as 30%. Because it was not possible to determine the genotype of every individual seedling, types and frequencies of phenotypes given in Table S6 should be interpreted cautiously.

### RT-PCR

Total RNA was isolated from leaf tissue using the RNeasy Plant Kit (Qiagen, Valencia, CA). 1 µg total RNA was used for cDNA synthesis and PCR, using a Superscript One-Step RT-PCR kit (Life Technologies, Grand Island, NY), and 35 cycles of amplification. See Table S4 for a list of the primers used. Reactions for each primer pair and mutant were compared to total RNA from wild-type plants, and to the expression of EF1α (At5g60390; FP CATGGGTGTTGGACAAACTT, RP CTCCTTGATGATTTCATCGT) [69].

### Enriched CO_2_ Screening

For comparing growth under photorespiratory and non-photorespiratory conditions, plants were grown in sets of 4 or 5 plants per 1 square inch pot or 16 plants per 2.5 square inch pot in duplicate flats in 0.3% CO_2_ and ambient air, with all other growth conditions as above. The photorespiratory mutant *shm1-1* [50] was grown in each flat as a control. To increase the chance of thorough sampling of the seed population, seeds were mixed well and examined in a dissecting microscope prior to sowing. For seed stocks with an obvious visual segregating seed phenotype, seeds were selected individually so that 25% abnormal and 75% normal appearing seeds were sown. Other controls used for growth in high CO_2_ and for chlorophyll fluorescence measurements include the photorespiratory mutants SALK_011035 of At5g04140, (Ferredoxin-dependent glutamate synthase 1; Fd-GOGAT), and SALK_073728, harboring a weak 5′-non-coding region allele of At1g80380 (encoding glycerate kinase).

### Chlorophyll Fluorescence Measurements

Chlorophyll fluorescence measurements were conducted on 14 day old seedlings as described previously [20], except plants were grown in 0.3% CO_2_. Dark adaptation, high light treatment, and chlorophyll fluorescence measurements were done in ambient air. Fv/Fm was measured for plants dark-adapted for 20 minutes, and NPQ was measured after treatment with 533 µmol photons/m^2^/s actinic light. Plants were subjected to high light stress (1,500–1,700 µmol photons/m^2^/s) for 3 hours, then Fv/Fm was measured as above immediately following high light treatment, and again after a 48 hours recovery period in 100 µmol photons/m^2^/s and 0.3% CO_2_. See Table S8 for a timeline summary. Cut-off values used for evaluating mutants were BHL Fv/Fm, 0.742+/−0.002; BHL NPQ, 0.285+/−0.0038, AHL Fv/Fm, 0.501+/−0.0038; Recovery Fv/Fm, 0.729+/−0.00. In every flat, in addition to the photorespiration controls described above, the At5g13050 (5-formyltetrahydrofolate cycloligase) mutant 5-FCL [62] was grown to confirm alterations in Fv/Fm fluorescence after high light treatment. The *npq1-2* mutant [70] was grown as a control for alterations in nonphotochemical quenching, and allele SALK_020654 of At3g56040 (UDP-glucose pyrophosphorylase 3) was grown to confirm alterations in *Fv/Fm* before high light treatment [71]. After measurements were made, all plants were genotyped to confirm that the observed phenotype was due to the presence of a homozygous mutation.

## Supporting Information

Figure S1 **Examples of mutants responsive to sucrose supplementation.** Seeds segregating for deleterious mutations were grown on medium with no sucrose (top panel of each section), 0.5% sucrose (middle panel each section) or 2% sucrose (bottom panel each section). Red arrows indicate individuals appearing to have the rescued homozygous lethal phenotype; large green and healthy plants are presumed heterozygous or wild type for the mutation. A and B are examples of classifications 4a and 4d from Figure 4, C and D are examples of classification 4b from Figure 4, and E and F are examples of classification 4c from Figure 4. A, allele SALK_002470 of At1g63970 encoding 2C-methyl-D-erythritol 2,4-cyclodiphosphate synthase; B, allele SAIL_810_G07 of At5g01590 encoding an unknown protein; C, allele SAIL_184_D06 of At1g68890, encoding a protein involved in phylloquinone biosynthesis; D, allele SALK_116713 of At5g63060 encoding a Sec14p-like phosphatidylinositol transfer family protein; E, allele SALK_151530 of At1g50900, encoding *GDC1*; F, allele SAIL_58_D02 of At3g60750 encoding a putative transketolase.
(TIFF)

Figure S2 **Examples of mutant responses to amino acid supplementation.** A and B, plants photographed directly on agar plates containing nutrient medium, red arrows indicate individuals judged to have the rescued homozygous lethal phenotype; vigorous plants are presumed heterozygotes or wild type for the mutation. Medium supplements: top, 0.5% sucrose, no amino acids; bottom, 0.5% sucrose, with amino acids. A, Mutant SALK_048976 of At1g09820, encoding a PPR protein. B, Chloroplast Calvin Cycle sedoheptulose-1,7-bisphosphatase gene (At3g55800) alleles SALK_090549, left, and SALK_130939, right. C, D, E: all individuals with the rescued homozygous lethal phenotype removed from the Petri plate for better visibility. The top individual in each panel (‘wt’) is a Col wild-type control plant from the same plate. C, allele SALK_151530 of At1g50900, encoding *GDC1*, left photograph, seedlings grown without amino acids or sucrose, right photograph, seedlings grown with amino acids, no sucrose). D, mutant allele SALK_015165 of At3g12290, encoding an amino acid dehydrogenase family protein, causes sensitivity to amino acid supplementation, left to right, no sucrose or amino acids, sucrose only, amino acids only, and sucrose with amino acids.
(TIFF)

Figure S3 **Examples of mutants that respond to growth in enriched CO_2_.** Left, ambient air; right, 0.3% CO_2_. Arrows indicate individuals with homozygous severe growth phenotype. A, top, wild-type Col; middle, homozygous *shm1-1* control; bottom, segregating population of allele SALK_044513 in At3g12590, encoding an unknown protein. B, top, wild type Col; bottom, allele FLAG_299H06 of At2g21380, annotated as a kinesin motor family protein.
(TIFF)

Figure S4 **Tryptophan mutants are rescued by amino acid supplementation.** A, Tryptophan synthase alpha subunit deficient mutant *trp3-1* [36] (At3g54640) 26 days after sowing. Upper, basal medium supplemented with 0.5% sucrose, lower, basal medium supplemented with 0.5% sucrose and amino acids. B, Tryptophan synthase beta subunit deficient mutant *trp 2-1* [37] (At5g54810) 22 days after sowing, upper, basal medium supplemented with 0.5% sucrose, lower, basal medium supplemented with 0.5% sucrose and amino acids.
(TIFF)

Table S1 **Confirmed and putative homozygous lethal mutants.**
(XLSX)

Table S2 **Mutants in genes likely to be non-essential under laboratory growth conditions.**
(XLSX)

Table S3 **All genotyping assays.**
(XLSX)

Table S4 **Gene expression for viable homozygous mutants.**
(XLSX)

Table S5 **Developing seed phenotypes for all mutants assayed.**
(XLSX)

Table S6 **Germination and growth on supplemented media for all mutants assayed.**
(XLSX)

Table S7 **Screen for growth in photorespiratory and non-photorespiratory conditions.**
(XLSX)

Table S8 **Chlorophyll fluorescence assay conditions.**
(DOCX)
