# Supplementary figures and images for: Analysis of Essential Arabidopsis Nuclear Genes Encoding Plastid-Targeted Proteins
Source: PLoS One. 2013 Sep 4;8(9):e73291. doi: 10.1371/journal.pone.0073291 (PMC3762728; doi:10.1371/journal.pone.0073291)

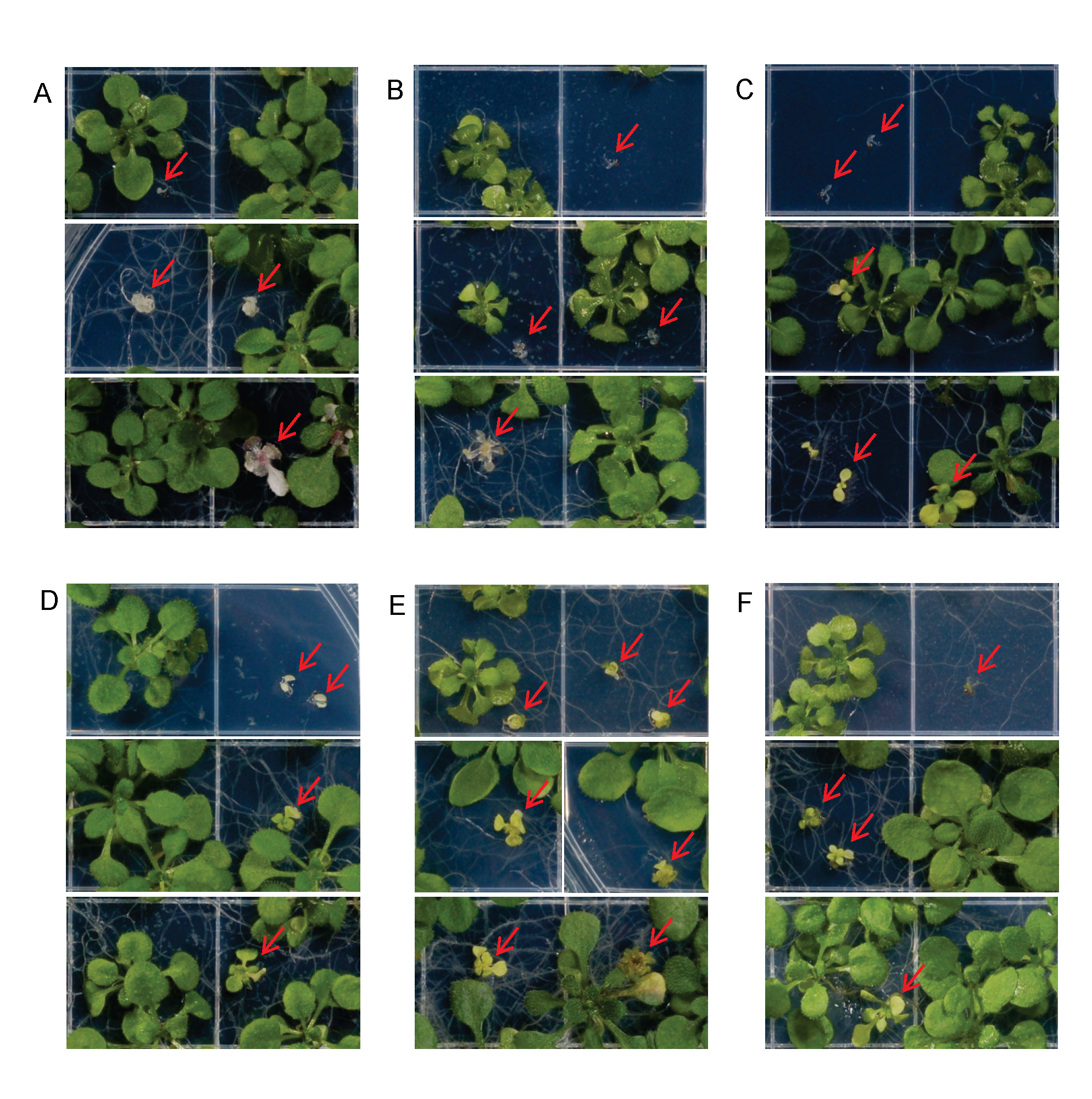

Supplement: Figure S1 — Examples of mutants responsive to sucrose supplementation. Seeds segregating for deleterious mutations were grown on medium with no sucrose (top panel of each section), 0.5% sucrose (middle panel each section) or 2% sucrose (bottom panel each section). Red arrows indicate individuals appearing to have the rescued homozygous lethal phenotype; large green and healthy plants are presumed heterozygous or wild type for the mutation. A and B are examples of classifications 4a and 4d from Figure 4, C and D are examples of classification 4b from Figure 4, and E and F are examples of classification 4c from Figure 4. A, allele SALK_002470 of At1g63970 encoding 2C-methyl-D-erythritol 2,4-cyclodiphosphate synthase; B, allele SAIL_810_G07 of At5g01590 encoding an unknown protein; C, allele SAIL_184_D06 of At1g68890, encoding a protein involved in phylloquinone biosynthesis; D, allele SALK_116713 of At5g63060 encoding a Sec14p-like phosphatidylinositol transfer family protein; E, allele SALK_151530 of At1g50900, encoding GDC1; F, allele SAIL_58_D02 of At3g60750 encoding a putative transketolase. (TIFF) [file pone.0073291.s001.tiff]

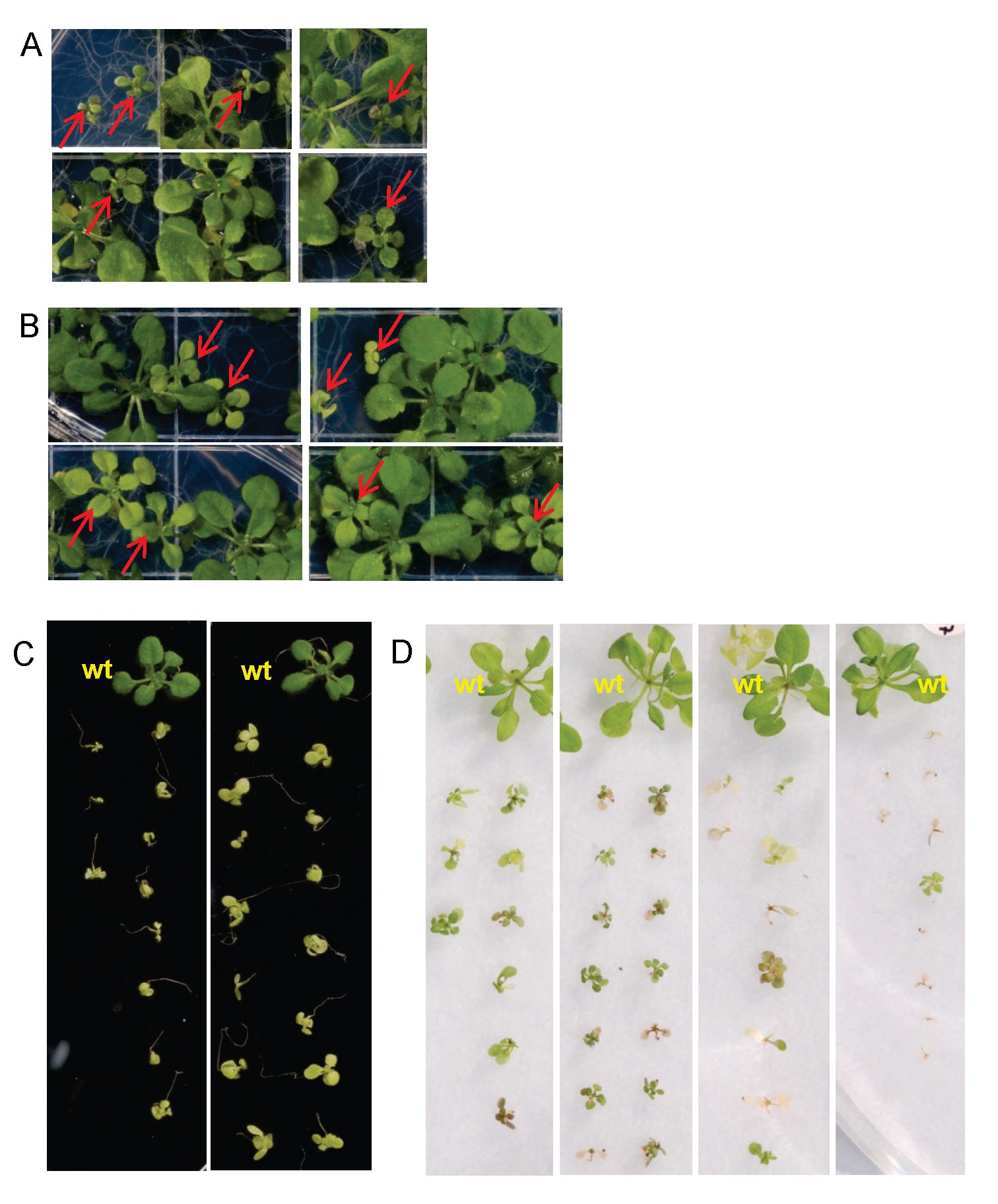

Supplement: Figure S2 — Examples of mutant responses to amino acid supplementation. A and B, plants photographed directly on agar plates containing nutrient medium, red arrows indicate individuals judged to have the rescued homozygous lethal phenotype; vigorous plants are presumed heterozygotes or wild type for the mutation. Medium supplements: top, 0.5% sucrose, no amino acids; bottom, 0.5% sucrose, with amino acids. A, Mutant SALK_048976 of At1g09820, encoding a PPR protein. B, Chloroplast Calvin Cycle sedoheptulose-1,7-bisphosphatase gene (At3g55800) alleles SALK_090549, left, and SALK_130939, right. C, D, E: all individuals with the rescued homozygous lethal phenotype removed from the Petri plate for better visibility. The top individual in each panel (‘wt’) is a Col wild-type control plant from the same plate. C, allele SALK_151530 of At1g50900, encoding GDC1, left photograph, seedlings grown without amino acids or sucrose, right photograph, seedlings grown with amino acids, no sucrose). D, mutant allele SALK_015165 of At3g12290, encoding an amino acid dehydrogenase family protein, causes sensitivity to amino acid supplementation, left to right, no sucrose or amino acids, sucrose only, amino acids only, and sucrose with amino acids. (TIFF) [file pone.0073291.s002.tiff]

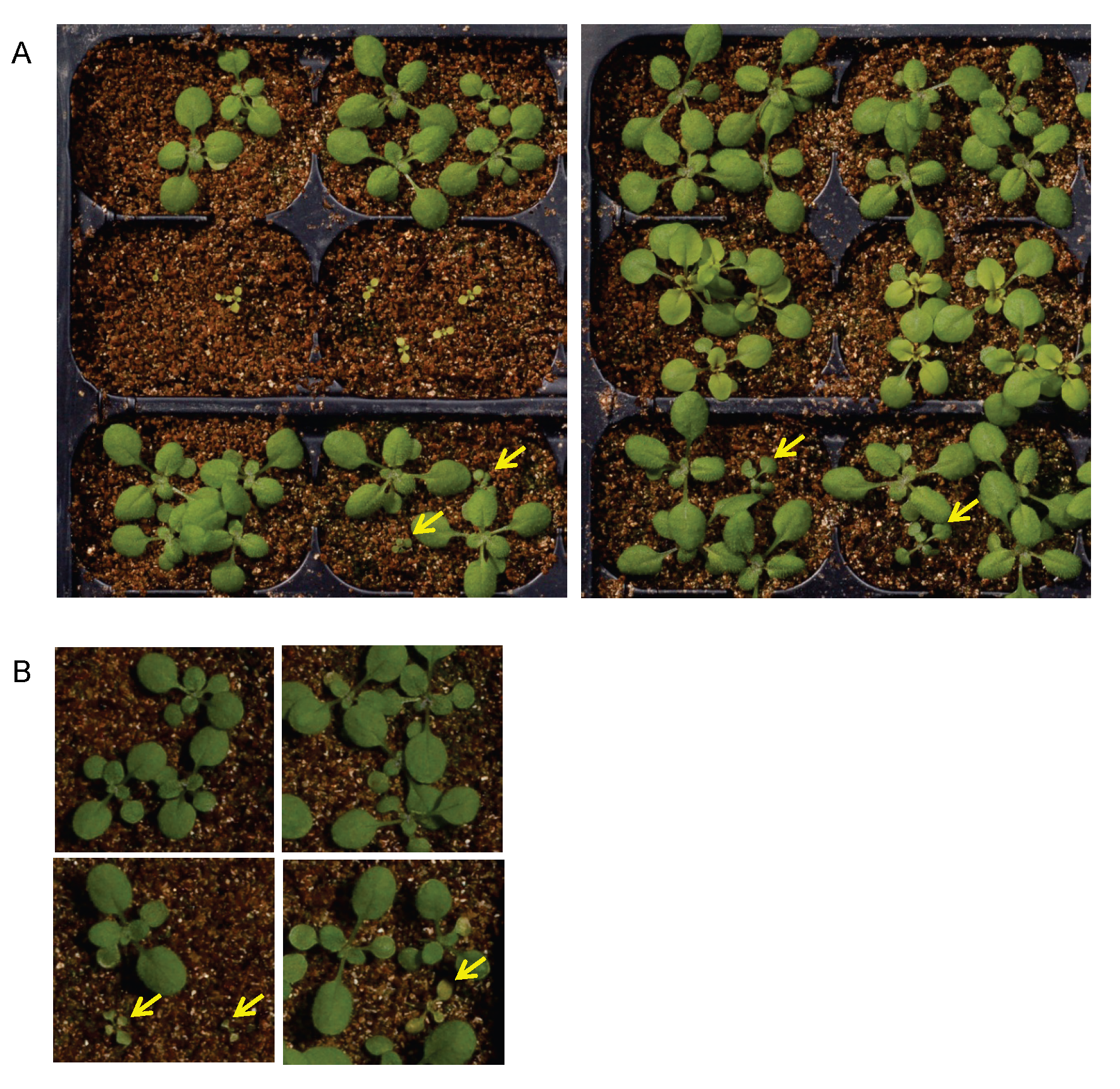

Supplement: Figure S3 — Examples of mutants that respond to growth in enriched CO2. Left, ambient air; right, 0.3% CO2. Arrows indicate individuals with homozygous severe growth phenotype. A, top, wild-type Col; middle, homozygous shm1-1 control; bottom, segregating population of allele SALK_044513 in At3g12590, encoding an unknown protein. B, top, wild type Col; bottom, allele FLAG_299H06 of At2g21380, annotated as a kinesin motor family protein. (TIFF) [file pone.0073291.s003.tiff]

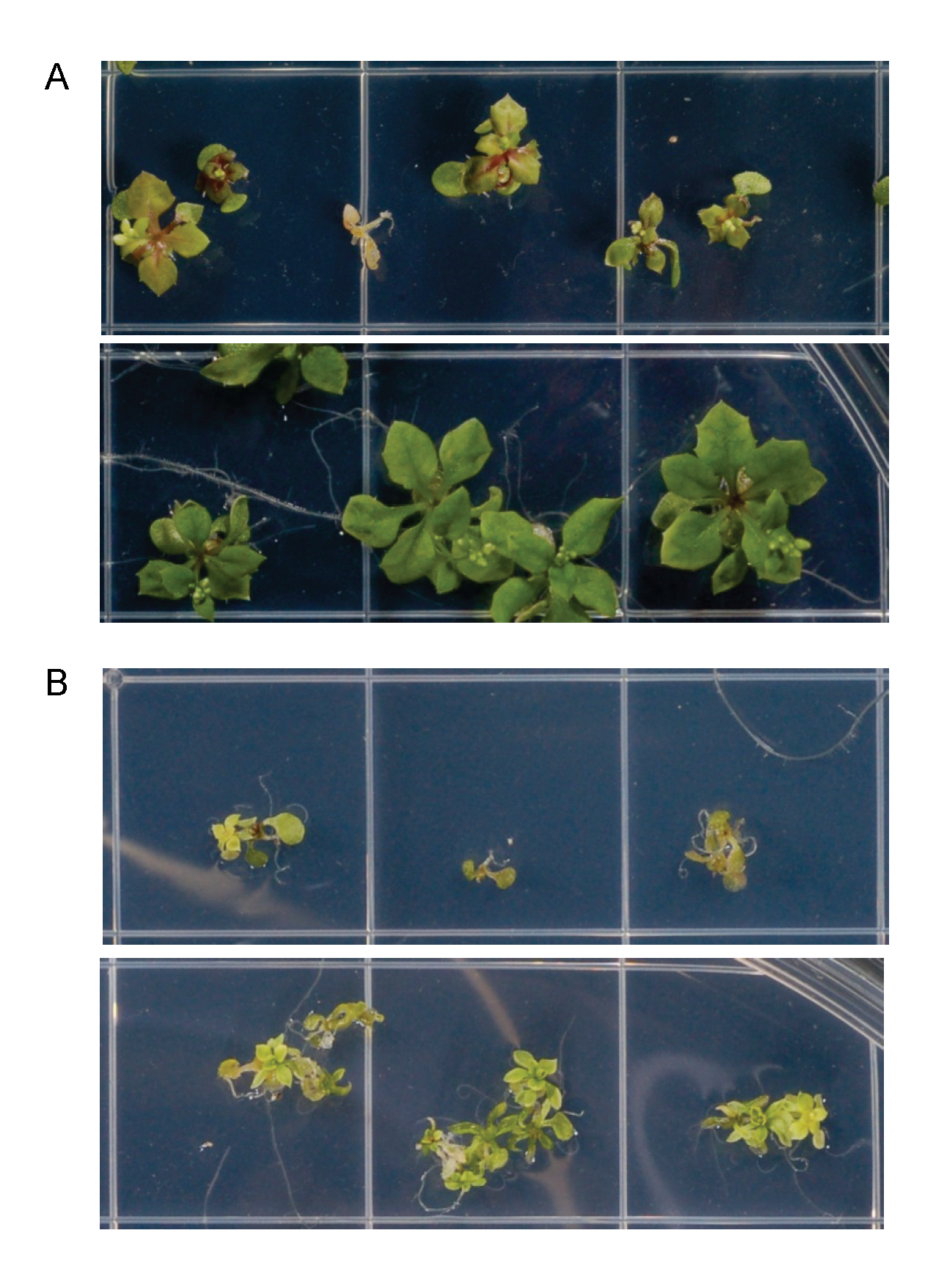

Supplement: Figure S4 — Tryptophan mutants are rescued by amino acid supplementation. A, Tryptophan synthase alpha subunit deficient mutant trp3-1 [36] (At3g54640) 26 days after sowing. Upper, basal medium supplemented with 0.5% sucrose, lower, basal medium supplemented with 0.5% sucrose and amino acids. B, Tryptophan synthase beta subunit deficient mutant trp 2-1 [37] (At5g54810) 22 days after sowing, upper, basal medium supplemented with 0.5% sucrose, lower, basal medium supplemented with 0.5% sucrose and amino acids. (TIFF) [file pone.0073291.s004.tiff]
