# Supplementary material for: Analysis of Essential Arabidopsis Nuclear Genes Encoding Plastid-Targeted Proteins
Source: PLoS One. 2013 Sep 4;8(9):e73291. doi: 10.1371/journal.pone.0073291 (PMC3762728; doi:10.1371/journal.pone.0073291)
Supplement: Table S8 — Chlorophyll fluorescence assay conditions. (DOCX) [file pone.0073291.s012.docx]

|  | Experiment timeline | | | | | | | | |
| --- | --- | --- | --- | --- | --- | --- | --- | --- | --- |
|  | Germination to 14 days | 20 min. | 20 min. | 3 hours | 20 min. | 20 min. | 2 days | 20 min. | 20 min. |
| Atmosphere | 0.3% CO2 | air | air | air | air | air | 0.3% CO2 | air | air |
| Light condition | 16 hours light/8 hours dark | Dark | Measuring lights | High light (1500 μmol photons/m^2^/s) | Dark | Measuring lights | 16 hours light/8 hours dark | Dark | Measuring lights |
| Fluorescence measurement |  |  | Fv/Fm BHL, NPQ BHL |  |  | Fv/Fm AHL |  |  | Fv/Fm Recovery |

Table S8. Chlorophyll fluorescence assay conditions.
